# Evaluation of College of Medicine Students’ Perception of a Subscription-Based Model of Primary Care Delivery

**DOI:** 10.7759/cureus.96784

**Published:** 2025-11-13

**Authors:** Justin G James, Erica Friedman, Daniel J Schlegel

**Affiliations:** 1 Family and Community Medicine, Penn State University College of Medicine, Milton S. Hershey Medical Center, Hershey, USA; 2 Medicine, Penn State University College of Medicine, Milton S. Hershey Medical Center, Hershey, USA

**Keywords:** accessible primary care, direct primary care, patient acceptance of healthcare, student healthcare, telehealth, virtual medicine

## Abstract

Background and objectives: Access to timely, affordable, and student-centered primary care remains a persistent challenge for college and graduate students, particularly those in demanding health professional programs. Traditional healthcare models often fall short in meeting students’ unique needs related to scheduling flexibility, geographic transitions during training, and financial constraints. To address these gaps, we developed COMMpanion, a subscription-based, virtual-first primary care model designed specifically for a graduate student population. This study evaluates student experiences with the COMMpanion model, including its feasibility, acceptability, and perceived effectiveness in improving access to care.

Methods: We conducted a descriptive, single-timepoint cross-sectional survey of users, analyzed with descriptive statistics only. An electronic self-report survey was distributed to all subscribers of COMMpanion at the Penn State College of Medicine who completed at least one visit. The survey assessed user experiences, usability, satisfaction, and perceived clinical outcomes.

Results: Of 114 enrolled patients, 42/114 (36.8% response rate) completed the survey. Most respondents were medical students 35/42 (83%) (mean age 27 years). Usability was rated highly: comfort discussing health virtually 41/42 (97%); concerns fully addressed 33/42 (79%); reduced delays in care 38/42 (90%). Compared with respondents’ prior in-person experiences, overall visit quality was rated better by 23/42 (55%) and the same by 16/42 (38%).

Conclusions: COMMpanion effectively addressed key barriers to healthcare access among College of Medicine students by providing an inexpensive, convenient, and acceptable alternative to traditional care. These findings support the feasibility of subscription-based virtual care models in academic settings and highlight the need for further research into their broader implementation, sustainability, and clinical impact.

## Introduction

Accessing primary care can be challenging, particularly for young adults navigating academic pressures, geographic transitions, inconsistent insurance coverage, and financial constraints [1,2]. This population, including college and graduate students, often experiences fragmented care, delayed treatment, and underuse of preventive services [3-5]. Traditional primary care models centered around brick-and-mortar clinics, limited hours, and fee-for-service billing frequently fail to meet the needs of students whose schedules, mobility, and budgets require greater flexibility [6,7]. As a result, many students forgo regular primary care altogether, instead relying on urgent care or emergency departments for issues better suited to longitudinal, preventive management [8,9].

In response to these access barriers, telehealth and subscription-based care models have emerged as promising alternatives to conventional care delivery [10-16]. Telehealth offers timely, remote access to providers, minimizing disruptions to academic and clinical responsibilities [17]. Meanwhile, subscription-based models such as direct primary care (DPC) operate outside of traditional fee-for-service insurance structures, offering patients a flat monthly fee for comprehensive, ongoing care [18]. These approaches can reduce administrative burdens, enhance continuity, and foster more personalized patient-provider relationships. When combined, virtual-first and subscription-based models may be uniquely positioned to meet the expectations of modern healthcare consumers, particularly students who value convenience, affordability, and reliability [19].

However, current implementations of these models are rarely optimized for institutionally affiliated or highly mobile populations such as students, trainees, or remote workers. Virtual care may be limited by the absence of in-person exams, potentially weakening patient-provider rapport [20,21]. Some DPC practices may rely heavily on in-person care visits or lack essential integration with health systems, limiting specialty referrals and coordination [22]. There remains a critical need for hybrid care models that are not only accessible and affordable but also integrated, continuous, and responsive to the specific needs of student populations.

To address these gaps, we developed COMMpanion, a subscription-based, virtual-first primary care service tailored to medical, physician assistant, and graduate students affiliated with the Penn State College of Medicine [23,24]. This program was implemented within the College’s academic health system, combining the flexibility of telehealth with the accessibility and comprehensiveness of a direct primary care framework. COMMpanion was designed to reduce financial and logistical barriers while providing continuity of care for a population at risk of falling through gaps in traditional healthcare delivery.

In this paper, we describe the development and implementation of the COMMpanion model and evaluate its early performance through a descriptive analysis of utilization data and student-reported experiences. Our goal was twofold: (1) to present the operational design and rationale for COMMpanion as a novel, student-focused care model and (2) to assess user impressions of its accessibility, quality, and value during its initial rollout. In doing so, we aim to inform ongoing efforts to redesign primary care delivery in ways that are cost-effective, convenient, and attuned to the lived realities of students in rigorous academic programs.

## Materials and methods

Survey design

We used a descriptive, single-timepoint, cross-sectional design to assess the feasibility and acceptability of the COMMpanion program. Eligible participants included all currently enrolled medical, physician assistant, and graduate students at Penn State College of Medicine who had opted into the COMMpanion program and completed at least one virtual visit with a provider during the pilot period (May 2023-May 2024). Of the 114 students who met these criteria, all were invited via email to complete a voluntary online survey. No random sampling or allocation was performed. The analytic sample consists of the 42 respondents who completed the survey (response rate: 36.8%). The evaluation was not intended or powered to assess clinical effectiveness. 

The survey instrument was adapted from previously published telehealth evaluation tools used in outpatient primary care and specialty settings [11,25,26]. A complete list of questions and response options is provided in Table 1. Items were reviewed by clinical and academic stakeholders for relevance to student populations and the COMMpanion model. Modifications included rewording for student-specific contexts and the addition of free-text fields to capture qualitative impressions. While the tool demonstrated face validity based on prior use, it was not psychometrically validated in this population. Reliability testing (e.g., Cronbach’s alpha) was not performed, as the survey was designed for exploratory quality improvement rather than hypothesis testing.

**Table 1:** COMMpanion Patient Questionnaire Gender, age, ethnicity, user campus location, name and year of user’s graduate program. Responses were required unless otherwise noted. † Optional free text included. †† Impressions regarding whether the patient saved money and time were also asked, with free responses asking to quantify and describe the savings.

Category	Question	Response Options
User Background	Do you currently have a primary care provider?	^† ^Yes, they are based in the Hershey area. Yes, they are NOT based in the Hershey area. I do not have a primary care provider
	Have you used telehealth for primary care before?	Yes, No
	Briefly describe the reason for your most recent telehealth visit:	^† ^First-Time Visit, Follow-Up Visit, Urgent Visit, Checkup
	What device did you use for your visit?	^†^ Smartphone, Tablet, Desktop Computer, Laptop Computer
	Where were you when you had your COMMpanion telehealth visit?	^†^ Home, Work/Clinical Site, Parked Automobile
Usability	It was easy for me to plan and receive confirmation for my visit through COMMpanion.	Strongly Disagree, Disagree, Neither Agree nor Disagree (Neutral), Agree, Strongly Agree
	It was easy for me to connect into my telemedicine visit.	
	I was satisfied with the video quality of the visit.	
	I was satisfied with the audio quality of the visit.	
	For the visit, I felt comfortable with the privacy on the provider's end of the conversation.	
	For the visit, I felt comfortable with the privacy on my end of the conversation.	
Clinical Experience	History… The ability of my doctor to understand my symptoms and problems through telehealth was...	Much Worse, Somewhat Worse, Stayed the Same, Somewhat Better, Much Better
	Connection… The personal connection with my provider during my telehealth visit was...	
	Plan of Care… The plan of care and provider recommendations by the end of the telehealth visit were…	
	Time Required… The amount of time my provider spent with me was...	
	Overall Quality… The overall quality of the telemedicine visit was...	
	Comfort Discussing Health Virtually… I felt comfortable talking about my healthcare needs with my provider during the telehealth visit.	Strongly Disagree, Disagree, Neither Agree nor Disagree (Neutral), Agree, Strongly Agree
	Perceived Provider Active Listening… I felt my provider paid attention and listened to me in my telehealth visit.	
	Concerns Addressed in Visit… My concerns could be fully addressed in a telehealth format, despite no in-person care.	
^†† ^General Impressions	I felt that my telehealth visit with COMMpanion reduced delays in the health care provided and received.	Strongly Disagree, Disagree, Neither Agree nor Disagree (Neutral), Agree, Strongly Agree
	^†^ I would use telemedicine/COMMpanion again for future primary care visits.	
	^†^ I felt that my telemedicine visit could have been better or improved.	

Data collection and analysis

A hyperlink to the survey created on REDCap was emailed to all COMMpanion users who had completed at least one visit. Invitations were sent twice weekly, every two weeks, from April 1, 2024, through May 28, 2024. Incomplete survey entries were excluded from the analysis (N=2, 5%). Respondent survey data were extracted from REDCap two weeks after the final invitation was sent on June 8, 2024.

Quantitative survey responses were analyzed using descriptive statistics to summarize user characteristics and experience metrics. Descriptive statistics and figures were generated using GraphPad Prism Version 10 (GraphPad Software, San Diego, CA).

Ethical review and reporting standards

This project was reviewed by the Penn State Institutional Review Board and determined to qualify as exempt research (STUDY#00024461). As such, informed consent was not obtained. The study adhered to the Revised Standards for Quality Improvement Reporting Excellence (SQUIRE 2.0) guidelines for design and reporting. Data will be made available by requesting access from the corresponding author.

## Results

COMMpanion model overview

In May 2023, COMMpanion was introduced as an optional, subscription-based primary care program offering unlimited virtual visits, secure messaging, and phone calls for a monthly fee shared as $5 paid by the student and $5 subsidized by the College of Medicine. The service was offered to approximately 640 medical and physician assistant students, and any graduate students who also had health insurance. Students were required to maintain health insurance for services beyond primary care (e.g., medications, lab tests, imaging, specialty consultations, and hospital care), which could be ordered by COMMpanion providers but for which COMMpanion did not submit any insurance claims.

Out of respect for existing continuity of care relationships, if students had an existing medical home, they were discouraged from leaving that primary care home in favor of COMMpanion. If they did opt in, they were generally discouraged from having a source of primary care outside of COMMpanion. In the absence of a dedicated student health clinic, COMMpanion functioned as the primary healthcare solution for the student population. However, students could seek care from other local providers, including those within and outside of the affiliated healthcare system. A separate health office served administrative and accreditation functions for the College of Medicine but did not provide clinical care.

Clinical services were delivered by a family medicine physician and a certified registered nurse practitioner, supported by two registered nurses and administrative staff. Telehealth services were available from 7 AM to 7 PM, Monday through Friday, via the AmWell platform. The electronic medical record system, Oracle/Cerner PowerChart, was used for documentation, chart review, prescribing, and placing test orders. Students could easily self-book appointments and communicate between visits through secure messages or phone calls via the AmWell portal. Although there was no in-person component of primary care delivery, providers could order tests and labs to be performed at any facility, including the affiliated medical center, which were billable at standard market rates and which the students could submit for insurance coverage. COMMpanion also facilitated specialty care support through e-consults, enabling providers to obtain management recommendations from specialists within 72 business hours at no additional cost to the patients.

COMMpanion user cohort characteristics

All respondents who completed the COMMpanion questionnaire were students at the Penn State College of Medicine Hershey campus who were enrolled in medicine (35, 83%), physician assistant (3, 7%), and PhD programs (4, 10%). Among the medical students who responded, 26 (74%) were first-years, 2 (6%) were second-years, 5 (14%) were third-years, and 2 (6%) were fourth-years. All physician assistant students were in their second year (3, 100%). Among the PhD students, one (25%) was in their first year, while three (75%) were in their third year. Among these participants, 19 (45%) were male and 23 (55%) were female. The ethnic distribution included White (21, 50%), Asian (13, 31%), Black (1, 2%), Hispanic/Latino (1, 2%), Middle Eastern (1, 2%), and other (5, 12%). The ages of the participants ranged from 22 to 45 years, with a mean age of 27 (SD ± 4) years.

When asked if they currently have a primary care provider (PCP), 12 (29%) respondents indicated they had a PCP in the local area, 14 (33%) had a PCP but not locally based, and 16 (38%) reported they did not have a PCP. Additionally, when queried about prior telehealth usage in primary care, a majority of respondents (25, 60%) indicated they had such experience, while 17 (40%) reported they did not.

Survey completion summary

**Figure 1:**
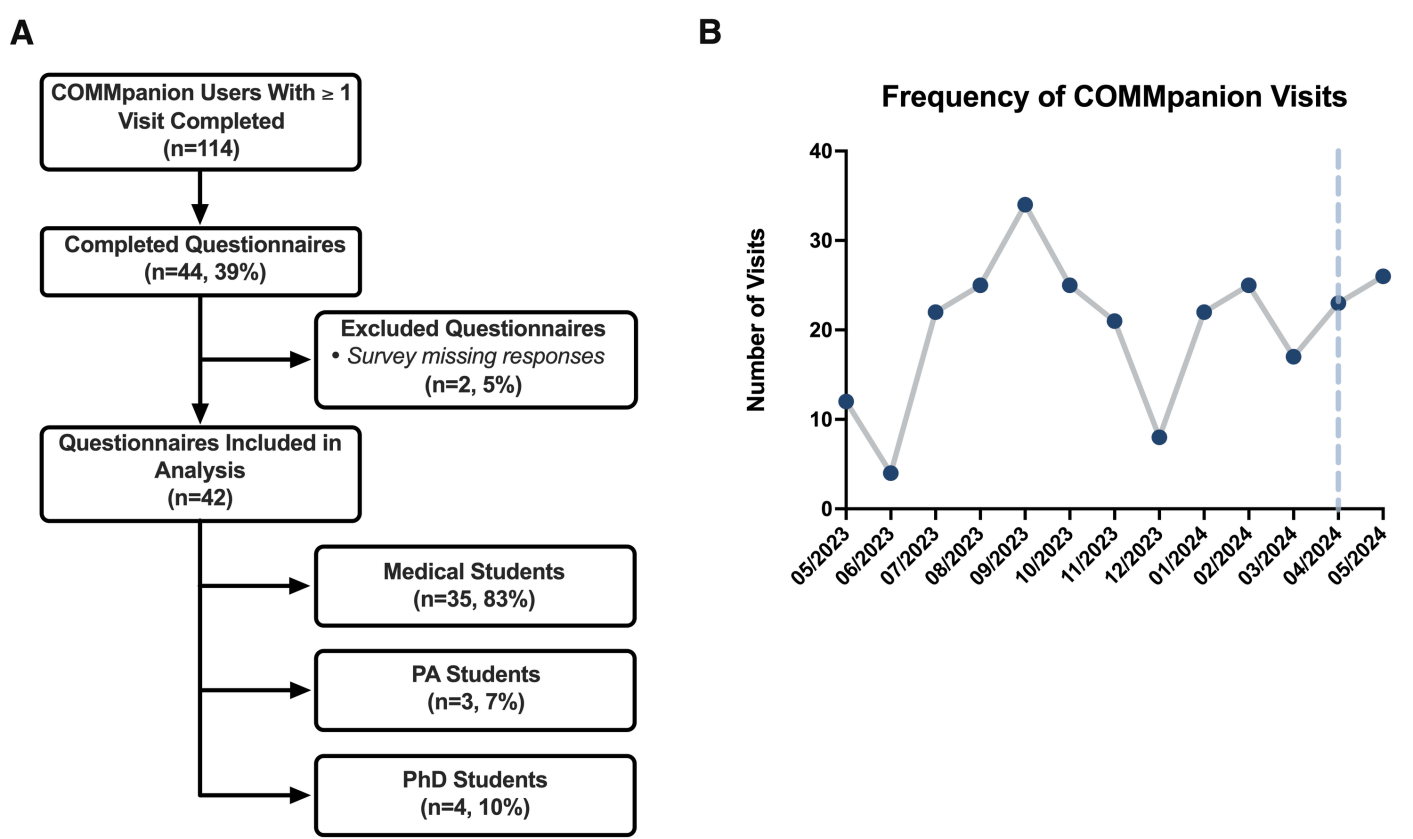
Survey Participation and Visit Utilization (A) Flowchart showing participant recruitment and response flow for the survey study, from eligible users with ≥1 COMMpanion visit (n=114) to final included respondents (n=42). (B) Number of COMMpanion visits per month from program launch to the end of the survey collection period. The dashed line marks the start of survey distribution. PA: Physician Assistant; PhD: Doctor of Philosophy.

COMMpanion users who completed the questionnaire between April 1 and June 8, 2024, were included in the formal analysis. From the beginning of the service in May 2023 to the end of survey collection, 264 virtual primary care visits were conducted, with 114 patients having at least one COMMpanion visit. A total of 44/114 (39%) surveys were completed. Two (5%) were excluded due to missing responses, leaving 42 (95%) completed surveys for analysis. A summary of the completed questionnaires and the frequency of COMMpanion visits are included in Figures 1A, 1B, respectively.

Visit summary and usability impressions

Respondents utilized COMMpanion for urgent visits (14, 33%), first-time visits to establish care (5, 12%), follow-up visits (13, 31%), check-ups (4, 10%), and a combination of the options mentioned earlier (6, 14%). Most respondents reported joining their virtual primary care visits through their laptop computers (22, 52%) or smartphones (19, 45%), with very few using a desktop computer (1, 2%) and none using a tablet (0, 0%). Patients were most likely to be at home (30, 71%), compared to at work or a clinical site (5, 12%) or in a parked automobile (1, 2%). Six (14%) respondents with more than one COMMpanion visit experience indicated that they joined their virtual primary care appointments from different locations each time, among the above-mentioned options.

The cohort overwhelmingly reported positive ratings of COMMpanion’s usability. The majority found it easy to plan and confirm visits (41, 98%) agreeing or strongly agreeing, connect to the telemedicine visit (42, 100%), and were satisfied with the video (42, 100%) and audio quality (42, 100%). Additionally, most respondents felt comfortable with the privacy from both the clinician’s end (41, 98%) and their end (41, 98%) during the conversation. Respondents believed they saved $56 and 60 minutes on average compared to routine care.

**Figure 2:**
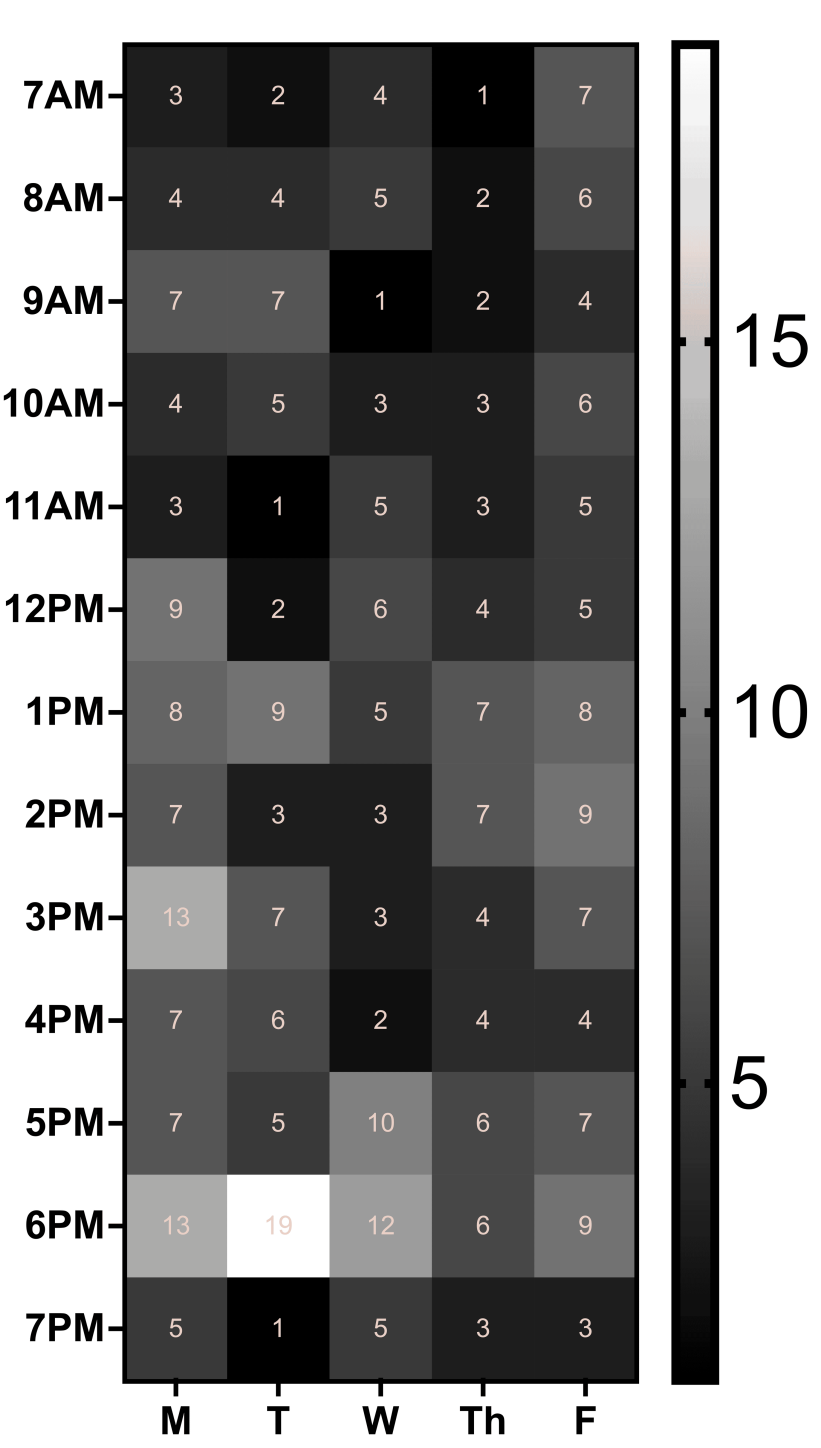
COMMpanion Service Utilization Service utilization patterns of the COMMpanion service over a five-day workweek (Monday through Friday) across hours of availability throughout the day (7 AM to 7 PM). The heatmaps depict the frequency of service use, with higher intensities (brighter colors) indicating peak utilization periods.

Utilization of COMMpanion peaked in the early and late afternoon, with lower activity observed during the early morning hours (Figure 2). Referral patterns were also analyzed separately. A total of 98 referrals for specialty care were made for 55 unique students. Of these, 85 (87%) were referrals to physicians and 13 (13%) were to non-physician services (Table 2).

**Table 2:** COMMpanion Specialty Referral Summary Summary of referrals to specialists for in-person care arranged through the COMMpanion service, listing the number and percentage of referrals made to various specialties, reflecting the total 98 referrals for specialty care made. The most frequent referrals were for gynecology (22%), dermatology (10%), and orthopedics (8%). Non-physician services accounted for 13% of all referrals, including physical therapy (7), audiology (2), neuropsychology (1), pelvic floor physical therapy (1), dietitian services (1), and nutrition services (1)

Specialty	N (%)
Allergy & Immunology	1 (1)
Breast Care	2 (2)
Cardiology	5 (5)
Colorectal	1 (1)
Dermatology	10 (10)
Endocrinology	1 (1)
Gastroenterology	6 (6)
General Surgery	4 (4)
Gynecology	22 (22)
Hematology	3 (3)
Medical Weight Loss/Obesity Medicine	1 (1)
Neurology	4 (4)
Neurosurgery	1 (1)
Ophthalmology	4 (4)
Orthopedics	8 (8)
Otolaryngology	4 (4)
Plastic Reconstructive Surgery	1 (1)
Psychiatry, Adult	1 (1)
Rheumatology	1 (1)
Sleep Medicine	2 (2)
Urology	3 (3)
Non-physician Services	13 (13)

Clinical experience and impressions

**Figure 3:**
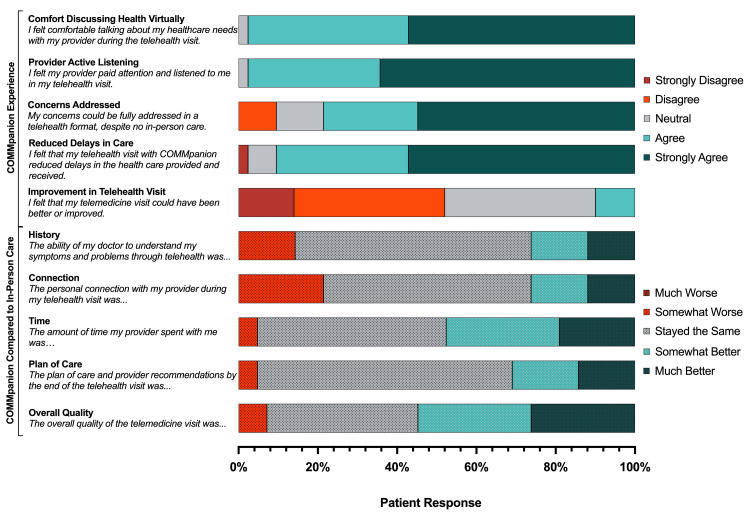
Summary of Patient Clinical Experiences Using COMMpanion Illustration summarizing patient impressions assessing the clinical components of their COMMpanion visit. Data reported as the percent of patients responding to each response category as represented by the colored, horizontal bar.

Respondents generally reported positive clinical experiences with the COMMpanion telehealth service for primary care. Most participants felt comfortable discussing their health virtually, with 57% (24) strongly agreeing and 40% (17) agreeing. Additionally, a significant proportion of respondents felt that their clinician actively listened (27, 64% strongly agreeing; 14, 33% agreeing) and that their concerns were fully addressed (23, 55% strongly agreeing; 10, 24% agreeing). Notably, 90% of respondents strongly agreed (24, 57%) or agreed (14, 33%) that their COMMpanion visit reduced delays in care. Overall, 62% (26) of respondents strongly agreed, and 29% (12) agreed they would use the COMMpanion service in the future. Over half of the respondents (6, 14% strongly disagreed; 16, 38% disagreed) reported that no improvements were necessary to their telehealth experience. A detailed breakdown of patients’ responses to their clinical experience using COMMpanion for virtual primary care is summarized in Figure 3.

Table 3 summarizes open-ended responses from COMMpanion users to free-text questions asked in the survey. The responses are categorized by thematic similarities, reflecting how patients felt they saved money, their reasons for future use of telemedicine, circumstances for preferring in-person visits, and suggestions for improving the telemedicine experience. Quoted user responses convey patient feedback and insight into their experiences and opinions.

**Table 3:** Summary of Open Responses from COMMpanion Users Summary of the open-ended responses provided in the patient questionnaire. The text responses were categorized by thematic and context similarities. Quoted user responses are included for each category.

Category Question	Response (Frequency, Percent)	Quoted User Responses
Saved Money: What ways do you believe you saved money using telemedicine?	Driving/Gas (24, 50%)	“Time from work/school, driving, gas, co-pay.” // “Time away from education.”
	Co-pay/bills (13, 27%)	
	Time from work/school (9, 19%)	
	Insurance savings (2, 4%)	
Future COMMpanion Use: I would use telemedicine/COMMpanion again for future primary care visits. Reason?	Efficiency/convenience of use and scheduling (13, 54%)	“I hope COMMpanion continues; this has tremendously improved my health. If I did not have COMMpanion, I likely would not be very good at following up with a doctor due to time alone and having an extremely busy schedule. Also, for some of the more acute things I needed, COMMpanion helped me avoid ER/Urgent Care for things they were able to prescribe me easily once I connected to the appointment.” // “Convenience of scheduling a same-day appointment from the comfort of my home. Is especially great that I don't have to drive somewhere while being sick.” // “My schedule is tough to work around as I can't easily flex it or take time off, so using COMMpanion is much easier and encourages me to stay on top of medical issues vs. scheduling an in-person visit”
	Accessibility to healthcare and medical orders (11, 46%)	
Circumstances for in-person care versus COMMpanion. Under what circumstances would you not use telehealth for a primary care visit and prefer an in-person visit?	In-person examination or testing needed; physical concern (18, 64%)	“If I needed GYN care, a physical exam, or mental health concerns.” // “I don't think I would use telehealth for serious physical concerns/illnesses/injuries because I felt that my care was significantly worsened since the provider could not see my appearance nor perform a physical exam.” // “I think I would likely utilize in-person care for urgent visits that would necessitate a physical or some testing. Sometimes it's hard to visualize and describe the sore throat the same way or explain a systematic illness over the visit- sometimes due to app limitations with being unable to exit the app during visits to take a picture (unless I am not using it correctly).” // “I would prefer an in-person visit for an acute problem that I would want a provider to have a hands-on look at, as I don't feel like telehealth would adequately convey my problem physically.”
	Difficulty describing/visualizing primary concern or symptoms (8, 29%)	
	Consult on sensitive personal health conversations (2, 7%)	
COMMpanion Visit Improvement: In what ways do you believe your telemedicine visit could have been better or improved?	Connectivity issues; audio/visual troubleshooting (2, 50%)	“If I get lab results before the telehealth visit." // "There were some issues with connectivity; the provider couldn't turn her video on, but that wasn't a problem.”
	Connect to specialized healthcare providers on demand (1, 25%)	
	Lab results tests available prior to telehealth visit (1, 25%)	

COMMpanion compared to in-person primary care

Finally, patients were asked to assess how COMMpanion compares to their previous experience with traditional, in-person primary care visits, such as the quality of the history obtained, connection with their clinician, time spent for the visit, plan of care, and overall quality of the telehealth visit. A detailed summary of the cohort’s responses is provided in Figure 3.

The majority of respondents reported that their experience with the COMMpanion virtual primary care service was comparable or better than their prior in-person visits. Specifically, 26% of respondents felt that their clinician’s ability to understand their symptoms and problems through telehealth was “much better” (5, 12%) or “somewhat better” (6, 14%) compared to an in-person visit, while 60% (25) felt it “stayed the same.” Regarding the personal connection with their clinician during the telehealth visit, 26% rated it as “much better” (5, 12%) or “somewhat better” (6, 14%), with 52% (22) stating it was unchanged compared to in-person care. Notably, of all the clinical components assessed, the personal connection with their clinician had the most negative impressions compared to an in-person visit, with 21% (9) of patients reporting it was somewhat worse. When it came to the amount of time the clinician spent with them, nearly half of the respondents (48%) felt it was either “much better” (8, 19%) or “somewhat better” (12, 29%), while another 48% (20) felt it stayed consistent with their in-person visits. For the plan of care and provider recommendations, 31% of respondents thought it was improved (6, 14% “much better”; 7, 17% “somewhat better”), with 64% (27) perceiving no change. Overall, 55% of respondents rated the quality of the telemedicine visit as either “much better” (11, 26%) or “somewhat better” (12, 29%) compared to their previous in-person experiences. In comparison, 38% (16%) perceived the quality was the same.

## Discussion

COMMpanion was developed to address a clear gap in student healthcare: the lack of accessible, inexpensive, and consistent primary care for young adults in academic settings. By combining telehealth delivery with a low-cost subscription-based payment structure, the model offered an alternative to insurance-dependent, in-person primary care that better aligned with the realities of student life. Students were able to access care from wherever they were - including from home, clinical rotations, or even parked cars - without perceived delays, administrative hurdles, or high out-of-pocket costs.

Importantly, 71% (N=30) of survey respondents reported not having a local primary care provider prior to enrolling in COMMpanion. For this group, COMMpanion filled a critical gap, enabling students to establish care relationships that might otherwise be delayed or foregone altogether. The service allowed students to connect with primary care from wherever they were-whether at home, on clinical rotations, or even sitting in parked cars, making healthcare more adaptable to their demanding and mobile lifestyles. This busy, mobile, and financially constrained population saved time and money without encountering significant technical or privacy concerns.

Most importantly, their COMMpanion clinical experiences were perceived as comparable to in-person care. The high frequency of repeat COMMpanion users could suggest that the care model is appealing. In turn, the model could serve as an acceptable means to support continuity of care over time, a hallmark of primary care. This interpretation is supported by generally positive scores for their clinician’s ability to understand their problems, establish a personal connection, and spend time with them.

Nonetheless, consistent with research showing that in-person exams enhance the provider-patient relationship, the lowest acceptability scores were related to the personal connection established during telehealth visits [27-29]. Despite being a concern of relatively high frequency, it was still only noted by about one-fifth of COMMpanion users.

While COMMpanion was designed as a fully virtual primary care model, user feedback highlighted the absence of in-person services as a relative dissatisfier. Several students expressed that having access to basic in-person capabilities, such as specimen collection for labs, diagnostic testing, or focused physical exams, could enhance the efficiency and perceived completeness of care. Notably, the most common referrals requested through COMMpanion visits were to gynecology, dermatology, and orthopedics-specialties that often depend on physical examination. These patterns suggest that certain referrals may have been avoidable if in-person capabilities were embedded within the model. To preserve its virtual-first nature, an expanded version of COMMpanion could incorporate in-person care selectively, initiated only after a telehealth visit and when deemed clinically necessary by the provider. These visits could focus on problem-based physical exams or specimen collection, coordinated through the COMMpanion platform. This hybrid approach may broaden the scope of services, reduce external referrals, and increase the model’s appeal to users who prefer a more flexible primary care experience.

This hypothesis-generating quality improvement study has several limitations. The population is not representative of the general population, with a mean age of 27 years and membership in an academic medical community. Comparisons were self-reported versus respondents’ own prior in-person experiences; no external comparator cohort was available. Given the voluntary nature of participation and a moderate response rate (36.8%), results are subject to self-selection bias, such as students who were more satisfied with their experience may have been more likely to respond. Importantly, this study did not assess clinical effectiveness outcomes such as reduced emergency department use, improved health metrics, or treatment adherence. All results reflect self-reported impressions, not objective health data. The survey instrument, while adapted from published tools, was not psychometrically validated in this population, and reliability testing was not performed. The analysis of free-text responses was descriptive and not based on a formal coding framework. Finally, we did not include a comparator group of students who chose not to use COMMpanion and instead relied on conventional, brick-and-mortar primary care services. Future studies should compare COMMpanion users with non-users to better understand relative differences in access, satisfaction, and engagement.

Nonetheless, given the general population’s potential interest in rapid access to telehealth-based primary care without limitations on the number and duration of visits, this model could appeal to a broader population. It is also worth noting that this self-selected population, being relatively young, opting into the program voluntarily, and with 60% (25) reporting prior telehealth experience, was likely predisposed to accept this unique care model and felt comfortable navigating the technical aspects of virtual care. Overall quality was reported as similar or better by most respondents. We did not assess perceptions of the service among College of Medicine students who did not choose to enroll. This study also did not report on the type of diagnoses managed or whether most visits were for acute or chronic problems. Future research should assess quality metrics, whether cost savings were objectively realized for patients or the sponsor-funder, and whether the service is financially sustainable.

## Conclusions

This report is the first to evaluate a student-focused, virtual-first, subscription-based model of primary care implemented within an academic health system. This study provides a unique window into the priorities, challenges, and expectations of young adult learners navigating complex academic demands while managing their healthcare needs. With strong usability, satisfaction, and indications of longitudinal engagement, COMMpanion represents a promising step toward modernizing primary care access for student populations.

Future implementations should be informed by the lived experiences of students, with attention to integrating select in-person services, ensuring financial sustainability, and expanding outreach to students who have not yet engaged with primary care services. Additionally, this care model may be adaptable to other populations, including students at other institutions, individuals in remote or underserved areas, and the general public. To support broader implementation, future research should rigorously evaluate clinical outcomes, cost effectiveness, and long-term sustainability, particularly in settings without institutional subsidies, and compare this care model with conventional approaches students use to access primary care, such as traditional fee for service and brick and mortar models.
